# A comparison of 10 accelerometer non-wear time criteria and logbooks in children

**DOI:** 10.1186/s12889-018-5212-4

**Published:** 2018-03-06

**Authors:** Eivind Aadland, Lars Bo Andersen, Sigmund Alfred Anderssen, Geir Kåre Resaland

**Affiliations:** 1 Department of Sport, Food and Natural Sciences, Faculty of Education, Arts and Sports, Western Norway University of Applied Sciences, Campus Sogndal, Box 133, 6851, Sogndal, Norway; 2 Department of Sports Medicine, Norwegian School of Sport Sciences, Box 4014 Ullevål Stadion, 0806, Oslo, Norway

**Keywords:** Objective measurement, Objective activity monitoring, Data reduction algorithms, Validity, Pediatric

## Abstract

**Background:**

There are many unresolved issues regarding data reduction algorithms for accelerometry. The choice of criterion for removal of non-wear time might have a profound influence on physical activity (PA) and sedentary time (SED) estimates. The aim of the present study was to compare 10 different non-wear criteria and a log of non-wear periods in 11-year-old children.

**Methods:**

Children from the Active Smarter Kids study performed 7-days of hip-worn accelerometer monitoring (Actigraph GT3X+) and logged the number of non-wear periods each day, along with the approximate duration and reason for non-wear. Accelerometers were analyzed using 10 different non-wear criteria: ≥ 10, 20, 30, 45, 60, and 90 min of consecutive zero counts without allowance for interruptions, and ≥60 and 90 min with allowance for 1 and 2 min of interruptions.

**Results:**

891 children provided 5203 measurement days, and reported 1232 non-wear periods ranging from 0 to 3 periods per day: on most days children reported no non-wear periods (77.1% of days). The maximum number of non-wear periods per day was 2 for the 90-min criterion, 3 to 5 for most criteria, 7 for the 20-min criterion, and 20 for the 10-min criterion. The non-wear criteria influenced overall PA (mean values across all criteria: 591 to 649 cpm; 10% difference) and SED time (461 to 539 min/day; 17% difference) estimates, especially for the most prolonged SED bouts. Estimates were similar for time spent in intensity-specific (light, moderate, vigorous, and moderate-to-vigorous) PA, but varied 6–9% among the non-wear criteria for proportions of time spent in intensity-specific PA (% of total wear time).

**Conclusions:**

Population level estimates of PA and SED differed between different accelerometer non-wear criteria, meaning that non-wear time algorithms should be standardized across studies to reduce confusion and improve comparability of children’s PA level. Based on the numbers and reasons for non-wear periods, we suggest a 45 or 60-min consecutive zero count-criterion not allowing any interruptions to be applied in future pediatric studies, at least for children older than 10 years.

**Trial Registration:**

The study is registered in Clinicaltrials.gov with identification number NCT02132494. Registered 7 April 2014.

**Electronic supplementary material:**

The online version of this article (10.1186/s12889-018-5212-4) contains supplementary material, which is available to authorized users.

## Background

Accelerometers have provided epidemiologists with an objective tool to measure movement, which overcomes many limitations by self-report measures. Still, there are many unresolved issues regarding data reduction algorithms and standardization to secure the highest possible data quality and consistency among studies [1, 2]. While choices related to the length of epochs used, criteria for hours to constitute a valid day and a valid week, and cut points deserve attention, the definition of non-wear time has received relatively little interest in the literature. As shown by Cain et al. [2], only half of pediatric studies applying accelerometry have reported the non-wear criterion used. This is surprising, as this criterion may have a profound influence on the data subsequently available for analyses [3–5] and on PA and SED estimates [4–9].

Non-wear time is the time during a measurement period where participants do not wear the accelerometer, and should be excluded from further analyses on the assumption that the remaining wear time is sufficiently representative for the whole measurement period. A challenge, however, given the considerable interest in sedentary behavior [10–13], is that the detection and removal of non-wear time is based on removing continuous time (consecutive epochs) of zero counts that may be easily confused with sedentary time. This confusion may lead to erroneous estimates of participants’ PA and SED.

The optimal non-wear criterion will likely vary with the population under investigation, due to different PA and SED patterns [8, 14–17], and possibly also with the type of accelerometer being applied. In children and youth, non-wear time definitions using Actigraph monitors vary from 10 to 180 min of consecutive minutes of zero counts, with 10 and 20-min criteria being the most used [2]. Variation is also seen in studies with adults, although a 60-min criterion allowing for 0–2 min of non-zero counts are frequently used [18–22]. However, according to a 24-h laboratory-based study, this criterion clearly overestimated non-wear time and underestimated SED in overweight-to-obese youth and adults [17]. Thus, the authors recommended a 90-min rather than a 60-min criterion. Although these findings were limited in terms of applicability to a free-living setting [23], several other studies in adults have recommended longer durations of the non-wear criterion being applied (90–180 min of consecutive zero counts with and without allowance of interruptions) [6, 24–26]. It should be noted that while these studies are based on different monitors (Actigraph 7164 and GT3X+, Actical, StepWatch), there seems to be a movement towards applying longer duration non-wear criteria to avoid misclassification of SED as non-wear time.

Based on 369–517 children aged 8–13 years Janssen et al. [8] compared non-wear criteria of 10, 20 and 60 min of consecutive zero counts against logs. They suggested a 20-min criterion being used in future studies, although the 20 and 60-min criteria performed similarly. This conclusion supports Esliger et al’s [27] suggestion that 20 min of consecutive zero counts is appropriate in children, as motionless bouts ≥20 min are biologically implausible. However, it has been suggested that this low threshold causes an unrealistic high number of non-wear periods, leading Chinapaw et al. [28] to suggest a 60-min criterion being used. Interestingly, the International Children’s Accelerometry Database (ICAD) [29], a large database pooling many child cohort studies using objective measurements of PA, apply a 60-min criterion allowing for 2 min of non-zero counts, which might be expected to result in a valid wear time somewhere in between of the previous recommendations. These conflicting recommendations and practices lead to confusion and reduced comparability among studies in pediatric epidemiology, and hinder knowledge development concerning the health promoting effects of physical activity.

The aim of the present study was to compare non-wear periods and wear time among 10 different accelerometer non-wear time criteria and a log during a 7-day measurement period in a large sample of 11-year-old children.

## Methods

### Subjects

We included 1129 fifth grade children from the Active Smarter Kids (ASK) cluster-randomized trial, conducted in Norway during 2014–2015, for the present analyses [30]. Physical activity was measured with accelerometry at baseline, mid-term and follow-up. At follow-up, children and their parents were asked to complete a logbook of non-wear periods during the 7-day measurement period. The children who provided valid accelerometer data and logbooks at follow-up were included in the present study.

Our procedures and methods conform to ethical guidelines defined by the World Medical Association’s Declaration of Helsinki and its subsequent revisions. The Regional Committee for Medical Research Ethics approved the study protocol. We obtained written informed consent from each child’s parents or legal guardian and from the responsible school authorities prior to all testing. The study is registered in Clinicaltrials.gov with identification number: NCT02132494.

### Procedures

We have previously published a detailed description of the study [30], but provide a brief overview of the relevant methods below. Physical activity was measured using the ActiGraph GT3X+ accelerometer (ActiGraph, LLC, Pensacola, Florida, USA). Children were instructed to wear the accelerometer on the right hip at all times for seven consecutive days, except during water-based activities or while sleeping. We analyzed all accelerometry data using the Kinesoft analytical software version 3.3.80 (KineSoft, Loughborough, UK), specifying 10 different non-wear criteria: ≥ 10, 20, 30, 45, 60, and 90 min of consecutive zero counts without any allowance for interruptions above zero counts, and ≥60 and 90 min of zero counts with allowance for 1 and 2 min of interruptions above zero counts (criteria 60–1, 60–2, 90–1, and 90–2). Data were restricted to hours 06:00 to 23:59, and a wear time criterion of ≥480 min/day as determined from the 10-min non-wear criterion. All analyses were based on accumulated data using 10-s epochs and a normal filtering option.

Outcomes for PA and SED for the different non-wear criteria were total PA (counts/min), SED (< 100 cpm), light PA (LPA) (100–2295 cpm), moderate PA (MPA) (2296–4011 cpm), vigorous PA (VPA) (≥ 4012 cpm), and moderate-to-vigorous intensity PA (MVPA) (≥ 2296 cpm) (min/day), as well as SED bouts of 5–9, 10–19, 20–29, 30–59, and ≥60 min of duration. We adopted previously established and validated cut points [31, 32] to define time in SED and PA.

Logbooks were a one-page sheet where children, with assistance from their parents, was asked to report the number of, duration of, and reason for non-wear periods lasting ≥10 min each day during the 7-day measurement period (Additional file 1: Figure S1). They were also asked to report the time they got up in the morning and the time for going to bed, which was used for estimating wear time. As applying a feasible log was our highest priority, in contrast to some previous studies [6, 33] we did not ask for reporting of specific start and stop times for the non-wear occurrences. Children were asked to remove the accelerometer during sleep. Thus, they were specifically asked to only report waking non-wear periods and not sleep non-wear periods. Logbooks were matched with accelerometer data, to allow for a day-by-day analysis of agreement between accelerometer-derived and self-reported number of non-wear periods. We included all logs that provided complete data for at least one day and at the same time provided valid accelerometer data.

### Statistics

We report descriptive statistics as means and standard deviations (SD), means and 95% confidence intervals (CI), or frequency distributions. Number of and time in SED bouts are reported as median and interquartile range (IQR) as these data were skewed. Further, we compared mean values and relationships between accelerometer estimates obtained by different non-wear criteria to assess the practical significance of applying the different non-wear criteria. We analyzed inter-relationships among PA and SED estimates as obtained by the 10 accelerometer non-wear criteria using Spearman’s rho (ρ), as some estimates were skewed.

All analyses were performed using IBM SPSS v. 23 (IBM SPSS Statistics for Windows, Armonk, NY: IBM Corp., USA).

## Results

### Children’s characteristics

The number of children that provided an accelerometer file available for analysis was 1115. Of these, 1004 children returned the logbook, of which 932 logbooks where possible to match with accelerometer data (i.e., date was provided). After removing days that did not fulfil the accelerometer wear time criterion of ≥480 min/day (907 days, 14% of total days) and days with missing logbook data (414 days, 7% of total days with valid accelerometer wear time), 5203 days from 891 children was left for analyses (Table 1).

**Table 1:** The children’s characteristics (*n* = 891)

	Boys (*n* = 453)	Girls (*n* = 438)
Age (years)	10.9 (0.3)	10.9 (0.3)
Body mass (kg)	39.2 (8.3)	40.0 (9.0)
Height (cm)	147 (7)	147 (7)
BMI (kg/m^2^)	18.1 (2.9)	18.4 (3.1)
Overweight/obese (%)	16/3	16/4
Physical activity level^a^		
Total PA (cpm)	633 (211)	566 (185)
SED (min/day)	523 (62)	528 (61)
LPA (min/day)	221 (35)	224 (36)
MPA (min/day)	44 (13)	37 (11)
VPA (min/day)	30 (16)	24 (12)
MVPA (min/day)	74 (26)	61 (20)

Values are means (SD). *BMI* body mass index, *PA* physical activity, *cpm* counts per minute, *SED* sedentary time, *LPA* light physical activity, *MPA* moderate physical activity, *VPA* vigorous physical activity, *MVPA* moderate-to-vigorous physical activity, ^a^All results are based on weekly means using the 60-min non-wear criterion

### Self-reported non-wear periods

**Fig. 1:**
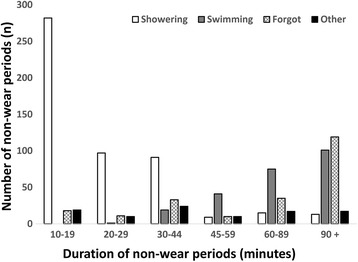
Reasons for non-wear periods of different durations (total *n* = 1098 non-wear periods)

Children reported a total of 1232 non-wear periods during waking hours on 22.9% of the days while under observation. For these days, the number of reported non-wear periods ranged from 1 to 3 periods per day (Table 2). Most reported non-wear periods were of 10–20 min duration (*n* = 333, 28.9%), whereas 123 (10.7%) periods were of 20–29 min duration, 175 (15.2%) periods were of 30–44 min duration, 75 (6.5%) periods were of 45–59 min duration, 153 (13.3%) periods were of 60–89 min duration, and 293 (25.4%) periods were of ≥90 min duration (data available for 1152 (93.5%) periods). Main reasons for not wearing the accelerometer was showering (46.2%), swimming (21.6%), and forgetting to wear it (20.6%), whereas 11.7% of non-wear periods were for other reasons (e.g., going to a party, playing football, climbing, or being sick or injured etc.) (data available for 1169 (94.9%) periods). Reasons for non-wear clearly differed across duration of the periods (Fig. 1); with more prolonged non-wear period durations showering decreased, whereas swimming and forgot to wear the accelerometer increased.

**Table 2:** Number of non-wear periods and wear time as reported in logs and analyzed by accelerometry using different non-wear criteria

	Number of non-wear bouts							Wear time
	Frequency (% of days)						Range	Mean (95% CI)
	0	1	2	3	4	5 +		
Logbook	*77.1*	*22.1*	*0.7*	*0.1*	*0.0*	*0.0*	*0*–*3*	*812 (809*–*814)*
Non-wear criteria^a^								
10	9.6	15.2	17.3	15.9	12.6	29.4	0–20	755 (753–758)
20	48.9	30.6	12.3	5.6	1.9	0.6	0–7	794 (792–797)
30	68.2	24.4	5.9	1.4	0.1	0.0	0–5	806 (803–808)
45	81.2	16.2	2.4	0.2	0.0	0.0	0–4	815 (812–818)
60	86.6	12.1	1.2	0.1	0.0	0.0	0–3	820 (818–824)
60–1	84.9	13.4	1.6	0.1	0.0	0.0	0–3	802 (800–805)
60–2	78.9	17.6	3.0	0.4	0.1	0.0	0–5	791 (788–794)
90	92.8	6.9	0.3	0.0	0.0	0.0	0–2	833 (830–836)
90–1	93.0	6.7	0.2	0.0	0.0	0.0	0–3	813 (810–816)
90–2	91.4	8.1	0.5	0.0	0.0	0.0	0–3	806 (803–808)

^a^Accelerometer non-wear criteria are minutes of consecutive zero counts without any allowance for interruptions above zero counts (10–90) and ≥60 and 90 min of consecutive zero counts with allowance for 1 and 2 min of interruptions above zero counts (60–1, 60–2, 90–1, and 90–2)

### Comparison of self-reported and accelerometer-determined non-wear periods

Number of non-wear periods per day as determined by accelerometry ranged from 0 to 20 across the non-wear criteria (Table 2), as opposed to 0–3 for the log. The 10-min non-wear criterion identified up to 20 non-wear periods per day, while the 20-min criterion resulted in up to 7 non-wear periods per day, and the other criteria resulted in 0 to 5 non-wear periods per day. Consistent with these results, wear time was lower for the strict criteria (e.g., 10 and 20-min criteria) than for the liberal criteria. Based on the number of non-wear periods, the 45, 60 and 60–1 accelerometer non-wear criteria compared most favorably with the log. Based on wear time, all of the 30, 45, 60, 90–1, and 90–2 differed less than ±10 min/day from the wear time estimated from the log.

Number of non-wear periods and wear time from different non-wear criteria differed minimally between boys and girls, and between normal weight and overweight/obese children (Additional file 2: Table S1).

### Physical activity and sedentary time for the different non-wear time criteria

**Fig. 2:**
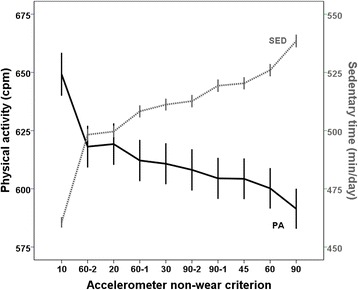
Mean (95% CI) sedentary time and overall physical activity level for the different non-wear criteria

Wear time differed by a maximum of 78 min per day (10%) between the non-wear criteria (Table 3). As expected, SED varied by a similar amount (78 min; 17%) (Fig. 2); the criterion resulting in the highest wear time gave the greatest amount of sedentary time, and vice versa. On the contrary, the criteria resulting in least wear time resulted in the greatest amount of overall PA, the greatest difference being 58 cpm (10%). Time spent in LPA, MPA, VPA, and MVPA did not differ across the non-wear criteria, whereas small differences were evident for proportions (6–9%) (Table 3).

**Table 3:** Wear time, overall PA level, and intensity-specific PA for the different non-wear criteria

Non-wear criteria^a^		SED	LPA	MPA	VPA	MVPA
	Wear time (minutes/day)	Time (minutes/day)				
10	755 (100)	461 (83)	224 (53)	40 (20)	27 (21)	67 (37)
20	794 (97)	500 (91)	224 (53)	40 (20)	27 (21)	67 (37)
30	805 (97)	511 (94)	224 (53)	40 (20)	27 (21)	67 (37)
45	815 (97)	520 (96)	224 (53)	40 (20)	27 (21)	67 (37)
60	820 (98)	526 (97)	224 (53)	40 (20)	27 (21)	67 (37)
60–1	802 (98)	508 (95)	224 (53)	40 (20)	27 (21)	67 (37)
60–2	791 (100)	498 (92)	224 (53)	40 (20)	27 (21)	67 (37)
90	833 (100)	539 (100)	224 (53)	40 (20)	27 (21)	67 (37)
90–1	813 (99)	519 (98)	224 (53)	40 (20)	27 (21)	67 (37)
90–2	805 (100)	513 (97)	224 (53)	40 (20)	27 (21)	67 (37)
	Overall PA (cpm)	Proportion of wear time (%)				
10	649 (339)	61.1 (8.2)	29.5 (5.4)	5.3 (2.5)	3.6 (2.8)	8.9 (4.7)
20	619 (327)	62.9 (8.5)	28.2 (5.7)	5.1 (2.4)	3.4 (2.6)	8.5 (4.6)
30	611 (323)	63.4 (8.5)	27.8 (5.8)	5.0 (2.4)	3.4 (2.6)	8.4 (4.5)
45	604 (321)	63.8 (8.5)	27.5 (5.8)	5.0 (2.4)	3.3 (2.6)	8.3 (4.5)
60	600 (319)	64.1 (8.5)	27.3 (5.8)	4.9 (2.4)	3.3 (2.6)	8.2 (4.5)
60–1	612 (326)	63.3 (8.6)	27.9 (5.8)	5.0 (2.4)	3.4 (2.6)	8.4 (4.6)
60–2	618 (329)	63.0 (8.5)	28.1 (5.8)	5.1 (2.4)	3.4 (2.7)	8.5 (4.6)
90	591 (315)	64.6 (8.5)	26.9 (5.8)	4.9 (2.4)	3.3 (2.6)	8.1 (4.4)
90–1	604 (323)	63.8 (8.6)	27.5 (5.9)	5.0 (2.4)	3.3 (2.6)	8.3 (4.5)
90–2	608 (325)	63.6 (8.6)	27.7 (5.9)	5.0 (2.4)	3.4 (2.6)	8.3 (4.5)

Values are means (SD). ^a^Accelerometer non-wear criteria are minutes of consecutive zero counts without any allowance for interruptions above zero counts (10–90) and ≥60 and 90 min of consecutive zero counts with allowance for 1 and 2 min of interruptions above zero counts (60–1, 60–2, 90–1, and 90–2); *PA* physical activity, *cpm* counts per minute, *SED* sedentary time, *LPA* light physical activity, *MPA* moderate physical activity, *VPA* vigorous physical activity, *MVPA* moderate-to-vigorous physical activity

Correlations for overall PA (ρ ≥ 0.99) and proportions of intensity-specific PA (ρ ≥ 0.93) were very high across all non-wear criteria. Correlations for SED were very high for proportions (ρ ≥ 0.94), but lower for absolute SED time (ρ ≥ 0.76). When excluding the most extreme non-wear criteria (i.e., the 10 and 90-min criteria), corresponding correlations were ρ ≥ 0.99, ρ ≥ 0.97, ρ ≥ 0.91, and ρ ≥ 0.98.

Sedentary time in bouts (number and minutes) was very similar between all non-wear criteria for bouts of 5 to 9 min of duration (Additional file 3: Table S2), correlations being ρ ≥ 0.98. However, differences increased substantially as the duration of the non-wear criteria started to interfere with the length of the bouts. The 10-min criterion clearly removed many 10-to-19-min bouts, the 10 and 20-min criteria removed many 20-to-29-min bouts, etc., leading to major differences across criteria for the longest bouts; the total number of SED bouts ≥30 and 60 min varied from 92 to 2730, and from 1 to 1023 bouts, respectively, across the 5203 measurement days. Thus, correlations were ρ ≥ 0.76, ρ ≥ 0.40, ρ ≥ 0.17, and ρ ≥ 0.03 among the criteria for SED bouts of 10–19 min, 20–29 min, 30–59 min, and ≥60 min, respectively.

## Discussion

We compared the number of waking non-wear periods and wear time among 10 different accelerometer non-wear criteria and logs of non-wear during a 7-day measurement period in 11-year-old children. According to the logs, the number of waking non-wear periods ranged from 0 to 3 periods per day; on most days children did not remove the accelerometer (77.1% of days). The choice of criterion caused up to 10% variation in population level estimates of SED and PA level after controlling for wear time. Yet, the practical significance of using different non-wear criteria for association analyses – where the rank of the children are important – are minor for criteria between 20 and 60-min, as correlations among estimates obtained from these criteria were high (ρ ≥ 0.97). The greatest differences among the criteria were found for estimates of SED in bouts of long durations. Determining wear and non-wear time will always be a classification-misclassification compromise, as SED and non-wear will be impossible to separate accurately using the current approach.

Previous studies have recommended defining child non-wear time as 20 to 60 min of consecutive zero counts (not allowing any time above zero counts) [8, 27, 28]. The recommendations by Esliger et al. [27] and Chinapaw et al. [28] were based on plausible values for length and number of non-wear periods, respectively. The current study together with the studies by Chinapaw et al. [28] and Toftager et al. [5] are rather consistent showing up to 10, 7, and 4 non-wear periods per day for 20, 30 and 60-min non-wear criteria, respectively. In comparison, we found 0–3 non-wear periods per day reported by the log. As concluded previously [28], we find it implausible that children remove the accelerometer more than 3–4 times per day on average. This conclusion is consistent with the current findings showing the best agreement between logbook and accelerometer estimates for the 45 (0–4 non-wear periods per day) and 60-min (0–3 non-wear periods per day) criteria. Yet, with increasing age, overall SED increase [34], the time spent in longer bouts of SED increase [8, 14, 15], and the number of breaks in SED time decrease [14, 16]. This development increase the risk to misclassify SED as non-wear time with increased age. This hypothesis is supported by a somewhat increased number of non-wear bouts across criteria in the study by Toftager et al. [5] in 11-to-14-year-olds and Chinapaw et al. [28] in 9-to-13-year-olds compared to the present study (11-year-olds), increased differences between SED estimates using more prolonged non-wear duration with older age [8, 9], and findings showing a greater overestimation of non-wear periods in adults compared to youth [17]. This supports the use of a 60-min criterion over a 45-min criterion in children older than 11-years old. Although the evidence for a relation of misclassification to other sociodemographic characteristics are conflicting [35, 36], such a relationship could lead to selective attrition due to violation of wear-time requirements, and result in skewed PA and SED estimates [5]. In any case, supporting a previous study [5], we do not recommend introducing group-specific non-wear criteria, as we believe such a practice easily could lead to more, rather than less, bias, in addition to increasing the complexity of data reduction procedures.

Criteria allowing some epochs above zero counts allows for touching or moving the accelerometer by accident and spurious spikes of accelerometer counts during non-wear without turning non-wear time into SED time [4]. Yet, allowing for interruptions might decrease classification accuracy [24] as well as making results vulnerable to variation in wear time if analyzed with different epoch lengths [37].

As it is impossible to accurately disentangle SED bouts from non-wear bouts, the applied non-wear criterion will be a trade-off, hopefully leading to the best possible classification-misclassification compromise. We found that showering was the main reason for not wearing the accelerometer for periods less than 30 min of duration (86.5%), whereas swimming in particular, but also forgetting to wear the accelerometer, and other reasons, were important reasons for periods over 30 min. Showering is not SED nor MVPA and would probably not have any meaningful influence on the data at a group or individual level. Thus, failing to remove these non-wear periods are arguable not critical. In contrast, failing to remove non-wear time spent swimming will clearly underestimate PA and overestimate SED, while we might hypothesize other activities might vary in intensity and thereby counterbalance each other. These findings suggest application of longer (more liberal) criteria over shorter (stricter) ones, supporting the 45 or 60-min criterion.

Consistent with previous studies [4–9], we found that the choice of non-wear time criterion affect population level estimates of PA and SED. Interestingly, the non-wear time criterion affects estimates of activity level to a much greater extent than other wear requirements (i.e., valid hours per day and valid days per week) [5]. Still, the non-wear criterion applied is the algorithm least frequently reported in the pediatric literature (missing in 48.6% of studies) [2]. We found a difference of up to 17% for SED time, whereas estimates for minutes of LPA, MPA, VPA or MVPA were not influenced by the non-wear criteria. Yet, overall PA (cpm) and proportions of intensity-specific PA varied up to 10% between criteria. Because wear time is most often controlled either as a covariate or by using percentages of valid wear time in analyses, variability is often introduced in estimates for all variables. Still, the high correlations among variables, especially for the 20-to-60 min non-wear time criteria, indicate that the non-wear criterion used probably have a minor impact in correlational designs, as previously shown in a large sample from the European Youth Heart Study [9]. Nevertheless, we strongly recommend future studies report non-wear time criteria along with other data reduction algorithms applied.

To the best of our knowledge, no previous studies have determined the influence of different non-wear time criteria on SED bouts. Contrary to findings for total SED and PA, we found great differences in estimates of prolonged SED bouts, probably being the most detrimental to health, if assuming that accumulation of SED in bouts were detrimental beyond total SED time. Giving non-wear criteria high attention therefore seems to be crucial in order to determine associations for SED bouts with health outcomes. Worth noting is that previous studies targeting associations between SED bouts and health in children have used a 20-min criterion [38, 39], a 60-min criterion [40], and a 60–2 criterion [41, 42]. Studies in adults have indicated that a longer non-wear criterion detects more time in prolonged bouts of SED [43, 44], however, such a pattern is not clear in child studies, possibly owing to other inconsistencies in bout definitions (e.g., whether the study allows for interruptions in SED within a bout) [38–42]. The present findings show that most criteria remove nearly all SED time in bouts over 60 min of duration, indicating that 11-year-old children are unable to sit uninterrupted for 60 min or more. Researchers investigating patterns of SED should be aware of this finding.

### Strengths and limitations

We consider the inclusion of 10 different accelerometer non-wear criteria together with a logbook for determining non-wear periods and wear time in a large representative sample of children strengths of the current study. In advance of some previous studies [5, 27, 28], the use of logs allows for determining agreement for different non-wear criteria, beyond reliance on common sense regarding plausible non-wear characteristics. Still, assessing behavior by self-report has several well-known limitations, possibly leading to bias in addition to general inaccuracy. For example, we might expect children to both forget and consciously underreport non-wear, but in contrast to a previous study in adolescents [33], we have no clear indication of underreporting. Additionally, because we did not assess specific time points for wear and non-wear in the log, we could not assess classification accuracy for each wear/non-wear period as determined by accelerometry.

We believe inclusion of results on non-wear periods as well as SED bouts for the different non-wear criteria are other strengths of the study. We suggest these measures be reported and considered “diagnostic” tools in future studies, to aid deciding whether the applied non-wear criterion seem sensible in a given study. Importantly, including such characteristics allow for qualified judgements about sensible criteria in large surveillance studies, without requirements for any additional data (e.g., logs). Developing new criterion measures, for example for time in and type of SED behavior [45], will be a clear advantage for establishing such standards.

While tri-axial accelerometry allows for detection of accelerations across three axes and might capture movement beyond a single axis, the present study was limited to apply the vertical axis only. Yet, a previous study has shown similar wear time based on vector magnitude and the vertical axis, indicating this issue being of minor importance [36].

## Conclusions

Based on a comprehensive evaluation of 10 different accelerometer non-wear criteria and logs to define number of non-wear periods and wear time in a large sample of 11-year-old children, we showed that population level estimates of PA and SED varied up to 10% among criteria. This finding shows that non-wear time algorithms should be standardized across studies to reduce confusion and improve comparability of children’s PA level. Yet, the practical significance of using different non-wear criteria for association analyses are minor for criteria between 20 and 60-min. We suggest a 45 or 60-min consecutive zero count-criterion not allowing any interruptions to be applied in future pediatric studies, at least for children older than 10 years. We recommend that future studies adapt a standardized approach for data reduction, until new small and waterproof devises that can be worn 24 h, or new approaches to improve accuracy of wear compliance-validation, based on for example touch, temperature or heart rate sensing, possibly become available for large-scale use in the foreseeable future [46].

## Additional files

Additional file 1: **Figure S1**. The logbook used for reporting of accelerometer non-wear periods. (PDF 526 kb)

Additional file 2: **Table S1**. Number of non-wear periods and wear time for the different non-wear criteria for girls, boys, normal weight and overweight/obese children. (DOCX 15 kb)

Additional file 3: **Table S2**. Number of SED bouts and time in SED bouts for the different non-wear criteria. (DOCX 15 kb)

## Abbreviations

BMI: Body mass index
CI: Confidence interval
CPM: Counts per minute
IQR: Interquartile range
LPA: Light physical activity
MPA: Moderate physical activity
MVPA: Moderate-to-vigorous physical activity
PA: Physical activity
SD: Standard deviation
SED: Sedentary time
VPA: vigorous physical activity
