# Supplementary material for: A comparison of 10 accelerometer non-wear time criteria and logbooks in children
Source: BMC Public Health. 2018 Mar 6;18:323. doi: 10.1186/s12889-018-5212-4 (PMC5840816; doi:10.1186/s12889-018-5212-4)
Supplement: Supplementary file 1 — Figure S1. The logbook used for reporting of accelerometer non-wear periods. (PDF 526 kb) [file 12889_2018_5212_MOESM1_ESM.pdf]

Name:

School:

Date of day number 1:

How many times did you remove your accelerometer today?

It is important to wear the accelerometer the whole waking day, but the unit should be removed when swimming and showering, and you might possibly forgot to put in back on after changing clothes? **In this log, we ask you to report every time you removed the accelerometer for more than 10 minutes. Please report such non-wear periods day-by-day. Please also report the duration of and the reason for the non-wear periods if you can remember. Also, please note the time you got up and went to bed.** As the accelerometer should be removed during sleep, DO NOT report sleep non-wear in this log.

Example

Got up: 07:10 **Went to bed: 20:50**

DAY 1: 1. time: ca. 90 minutes (swimming during school hours), 2. time: ca. 30 minutes (forgot to wear)

DAY 1: **Got up:** **Went to bed:**

____

DAY 2: **Got up:** **Went to bed:**

____

DAY 3: **Got up:** **Went to bed:**

____

DAY 4: **Got up:** **Went to bed:**

____

DAY 5: **Got up:** **Went to bed:**

____

DAY 6: **Got up:** **Went to bed:**

____

DAY 7: **Got up:** **Went to bed:**

____

Thank you for your reporting!
