# Supplementary material for: A comparison of 10 accelerometer non-wear time criteria and logbooks in children
Source: BMC Public Health. 2018 Mar 6;18:323. doi: 10.1186/s12889-018-5212-4 (PMC5840816; doi:10.1186/s12889-018-5212-4)
Supplement: Supplementary file 2 — Table S1. Number of non-wear periods and wear time for the different non-wear criteria for girls, boys, normal weight and overweight/obese children. (DOCX 15 kb) [file 12889_2018_5212_MOESM2_ESM.docx]

**Table S1**. Number of non-wear periods and wear time for the different non-wear criteria for girls, boys, normal weight and overweight/obese children.

|  | **Girls (2560 days)** | | **Boys (2576 days)** | | **Normal weight (3951 days)** | | **Overweight/obese (1064 days)** | |
| --- | --- | --- | --- | --- | --- | --- | --- | --- |
|  | **Non-wear periods (range)** | **Wear time (mean)** | **Non-wear periods (range)** | **Wear time (mean)** | **Non-wear periods (range)** | **Wear time (mean)** | **Non-wear periods (range)** | **Wear time (mean)** |
| **Logbook** | *0-3* | 810 | *0-3* | 814 | *0-3* | 811 | *0-2* | 817 |
| **Non-wear criteria*** |  |  |  |  |  |  |  |  |
| **10** | 0-18 | 755 | 0-20 | 754 | 0-20 | 755 | 0-18 | 750 |
| **20** | 0-7 | 793 | 0-7 | 795 | 0-7 | 793 | 0-7 | 795 |
| **30** | 0-4 | 804 | 0-5 | 806 | 0-4 | 805 | 0-5 | 807 |
| **45** | 0-3 | 813 | 0-4 | 816 | 0-4 | 814 | 0-3 | 816 |
| **60** | 0-3 | 819 | 0-3 | 822 | 0-3 | 820 | 0-3 | 822 |
| **60–1** | 0-3 | 801 | 0-3 | 802 | 0-3 | 801 | 0-2 | 803 |
| **60–2** | 0-4 | 791 | 0-5 | 790 | 0-5 | 790 | 0-3 | 791 |
| **90** | 0-2 | 830 | 0-2 | 835 | 0-2 | 832 | 0-2 | 835 |
| **90–1** | 0-2 | 812 | 0-3 | 814 | 0-3 | 812 | 0-2 | 815 |
| **90–2** | 0-2 | 805 | 0-3 | 806 | 0-3 | 804 | 0-2 | 807 |

*Accelerometer non-wear criteria are minutes of consecutive zero counts without any allowance for interruptions above zero counts (10-90) and ≥ 60 and 90 minutes of consecutive zero counts with allowance for 1 and 2 minutes of interruptions above zero counts (60–1, 60–2, 90–1, and 90–2).
