# Supplementary material for: A comparison of 10 accelerometer non-wear time criteria and logbooks in children
Source: BMC Public Health. 2018 Mar 6;18:323. doi: 10.1186/s12889-018-5212-4 (PMC5840816; doi:10.1186/s12889-018-5212-4)
Supplement: Supplementary file 3 — Table S2. Number of SED bouts and time in SED bouts for the different non-wear criteria. (DOCX 15 kb) [file 12889_2018_5212_MOESM3_ESM.docx]

**Table S2** Number of SED bouts and time in SED bouts for the different non-wear criteria.

|  | **Sedentary bout definition (min)** | | | | |
| --- | --- | --- | --- | --- | --- |
| **Non-wear criteria*** | **5–9** | **10–19** | **20–29** | **30–59** | **≥ 60** |
|  | **Number of bouts (bouts/day)**** | | | | |
| **10** | 14 (8) | 2 (3) | 0 (0) | 0 (0) | 0 (0) |
| **20** | 14 (8) | 5 (4) | 0 (0) | 0 (0) | 0 (0) |
| **30** | 14 (8) | 5 (4) | 1 (1) | 0 (0) | 0 (0) |
| **45** | 14 (8) | 5 (4) | 1 (1) | 0 (1) | 0 (0) |
| **60** | 14 (8) | 5 (4) | 1 (1) | 0 (1) | 0 (0) |
| **60–1** | 14 (8) | 4 (4) | 0 (1) | 0 (1) | 0 (0) |
| **60–2** | 13 (7) | 4 (4) | 0 (1) | 0 (0) | 0 (0) |
| **90** | 14 (8) | 5 (4) | 1 (1) | 0 (1) | 0 (0) |
| **90–1** | 14 (8) | 4 (4) | 1 (1) | 0 (1) | 0 (0) |
| **90–2** | 14 (8) | 4 (4) | 0 (1) | 0 (1) | 0 (0) |
|  | **Number of bouts (total)** | | | | |
| **10** | 75548 | 14773 | 877 | 92 | 1 |
| **20** | 73657 | 26692 | 2688 | 543 | 6 |
| **30** | 73429 | 26496 | 4714 | 960 | 15 |
| **45** | 73353 | 26437 | 4685 | 2207 | 63 |
| **60** | 73342 | 26418 | 3788 | 1345 | 115 |
| **60–1** | 72536 | 25495 | 4149 | 1672 | 5 |
| **60–2** | 71505 | 24607 | 4623 | 2730 | 1 |
| **90** | 73312 | 26393 | 4656 | 2711 | 1023 |
| **90–1** | 72664 | 25722 | 4314 | 1973 | 474 |
| **90–2** | 72104 | 25361 | 4162 | 1788 | 391 |
|  | **Time in bouts (min/day)**** | | | | |
| **10** | 96 (53) | 32 (38) | 0 (0) | 0 (0) | 0 (0) |
| **20** | 93 (52) | 62 (58) | 0 (0) | 0 (0) | 0 (0) |
| **30** | 93 (52) | 62 (57) | 21 (28) | 0 (0) | 0 (0) |
| **45** | 93 (52) | 62 (57) | 21 (28) | 0 (33) | 0 (0) |
| **60** | 93 (52) | 62 (57) | 21 (28) | 0 (37) | 0 (0) |
| **60–1** | 92 (52) | 58 (56) | 0 (26) | 0 (30) | 0 (0) |
| **60–2** | 90 (52) | 57 (55) | 0 (26) | 0 (0) | 0 (0) |
| **90** | 93 (52) | 62 (57) | 21 (28) | 0 (37) | 0 (0) |
| **90–1** | 92 (52) | 60 (56) | 20 (27) | 0 (32) | 0 (0) |
| **90–2** | 91 (52) | 58 (56) | 0 (26) | 0 (31) | 0 (0) |

*Accelerometer non-wear criteria are minutes of consecutive zero counts without any allowance for interruptions above zero counts (10-90) and ≥ 60 and 90 minutes of consecutive zero counts with allowance for 1 and 2 minutes of interruptions above zero counts (60–1, 60–2, 90–1, and 90–2). **Values are medians (IQR).
